# A new technique of autologous bone grafting for open-wedge high tibial osteotomy

**DOI:** 10.3389/fsurg.2024.1337668

**Published:** 2024-03-05

**Authors:** Yongchao Gong, Lin Jin, Yanwei Wang, Boxu Liu, Pengfei Shen, Zhiang Zhang, Peizhi Yuwen, Kuo Zhao, Lijie Ma, Wei Chen, Yingze Zhang

**Affiliations:** ^1^ Department of Orthopaedic Surgery, Third Hospital of Hebei Medical University, Shijiazhuang, Hebei, China; ^2^ Department of Orthopaedic Surgery, North China Medical and Health Group Xingtai General Hospital, Xingtai, Hebei, China

**Keywords:** open-wedge high tibial osteotomy, autologous bone grafting, osteotomy gap, proximal fibula osteotomy, fibula transplantation

## Abstract

**Purpose:**

This study aimed to demonstrate the application of orthotopic bone flap transplantation with a fibula transplantation (OBFT-FT) in open-wedge high tibial osteotomy (OW-HTO) and to assess the effect of OBFT-FT on gap healing.

**Patients and methods:**

From January to July 2020, 18 patients who underwent OW-HTO with OBFT-FT were reviewed for this study. Demographics, postoperative complications, and radiological and clinical outcomes of patients were collected. Finally, the clinical outcomes of patients were analyzed.

**Results:**

A total of 14 patients were included in this study. The average age and body mass index were 59.6 ± 9.2 years and 28.1 ± 4.5 kg/m^2^, respectively. The average correction angle and gap width were 9.5 ± 1.8° and 10.2 ± 2.7 mm, respectively. The rates of radiological gap healing at sixth week, third month, and sixth month were 42.9%, 85.7%, and 100%, respectively. The mean Lysholm score, International Knee Documentation Committee score, and visual analog scale scores at sixth-month follow-up were significantly better than the preoperative scores (*p* < 0.001, *p* < 0.001, *p* = 0.001, respectively). And, no delayed union or non-union, collapse, loss of correction, or surgical site infection were found.

**Conclusions:**

As a new technique for autologous bone graft, the OBFT-FT could be successfully applied in the treatment of gap healing after OW-HTO, and excellent radiological and clinical outcomes could be seen on patients’ short-term follow-up.

## Introduction

High tibial osteotomy (HTO) is a reliable surgical option for patients with medial compartment knee joint osteoarthritis (MCKOA) and varus deformity (1–4). Currently, medial open-wedge high tibial osteotomy (OW-HTO) is more popular than the lateral closed-wedge high tibial osteotomy (CW-HTO) for providing better bone stock preservation, more predictable and adjustable correction, and more minimally invasive surgical exposure, damage to the proximal tibiofibular joint, fibular nerve palsy, and so on (5, 6). However, osteotomy gap is an inevitable disadvantage of the OW-HTO causing delayed union, non-union, collapse, and loss of correction (7). It was reported that the mean time for patients to obtain radiological bone healing without bone graft ranges from 3 to 7.2 months (8–11). To accelerate the healing of the osteotomy gap, various gap fillers are used, such as autologous bone graft, allogeneic bone graft, and graft substitutes (12).

Autologous bone grafting harvested mainly from the iliac crest graft is currently recognized as one of the most effective and popular methods for the healing of osteotomy gap with its osteogenic, osteoinductive, and osteoconductive capacities (7, 13). But it has been reported that harvesting morbidities can range from 0.76% to 26%, and significant challenges are presented in terms of prolonged pain, increased bleeding, and operation time. Allograft can offer osteoconductive properties without donor site morbidities, but it lacks osteogenic properties and results in slower bone union (14). In addition, it has the disadvantages of requiring specialist storage, carrying with it a donor requirement, and having the potential risk of developing surgical site infection, immunologic reaction, or disease transmission (15, 16). Some surgeons prefer to use synthetic bone substitutes, although the disadvantages of synthetic bone substitutes including increased costs and increased risks of delayed union or non-union cannot be ignored (7).

Proximal fibular osteotomy is an effective method for treatment of early knee osteoarthritis (17–19). In addition, it has been confirmed that the pain relief and improvement are statistically significant (20). Due to fewer complications and easy operation, proximal fibular osteotomy is recommended in any treatment algorithm for MCKOA (21, 22). Meanwhile, the excised fibula serves as an ideal gap filler for filling the osteotomy gap in OW-HTO. This technique presents excellent biological properties and provides stable mechanical support.

The question regarding osteotomy gap fillers in OW-HTO that still exists is whether there is one material that can offer faster bone union and ensure less pertinent adverse events. To enhance the healing of the osteotomy gap and improve initial mechanical stability, we propose a new technique called orthotopic bone flap transplantation with a fibula transplantation (OBFT-FT) in OW-HTO. The purposes of this study were to demonstrate the application of OBFT-FT in OW-HTO and to assess its effect on gap healing.

### Patients and methods

This retrospective study was carried out in a tertiary university hospital. From January to July 2020, 18 patients who underwent OW-HTO with OBFT-FT in our department were included. The approval of the Institutional Review Board of the Hebei Medical University Third Affiliation was obtained, and the consent of all patients was obtained. The inclusion criteria were as follows: (1) stable knee with MCKOA in a varus morphotype requiring OW-HTO; and (2) OBFT-FT was applied in OW-HTO. The exclusion criteria were as follows: (1) Ahlbäck grade >3, (2) varus alignment >15° varus, (3) flexion contracture ≥15° or further flexion ≤90°, (4) lateral compartment arthritis or patellofemoral arthritis, and (5) patients lost to follow-up.

After excluding 4 patients according to the inclusion and exclusion criteria, 14 patients (14 knees) were ultimately included in this study. The patient demographic data and radiographic data are presented in Table 1. The average age and body mass index (BMI) were 59.6 ± 9.2 years and 28.1 ± 4.5 kg/m^2^, respectively. The average correction angle and gap width were 9.5 ± 1.8° and 10.2 ± 2.7 mm, respectively. Lateral hinge fracture was defined as discontinuity or a callus on the lateral cortex of the osteotomy site, and the incidence of it was 7.1%.

**Table 1:** Patient demographic data and radiographic data.

Variables	Number
Age (years), mean ± SD	59.6 ± 9.2
Gender (male), no. (%)	3 (21.4)
BMI, mean ± SD	28.1 ± 4.5
Correction angle (°), mean ± SD	9.5 ± 1.8
Gap width (mm), mean ± SD	10.2 ± 2.7
Lateral hinge fracture, *n* (%)	1 (7.1)

### Surgical technique

The patient is placed supine on a radiolucent operating table. After anesthesia, routine skin preparation and draping were performed and the proximal femoral tourniquet was inflated to 280 mmHg.

**Figure 1:**
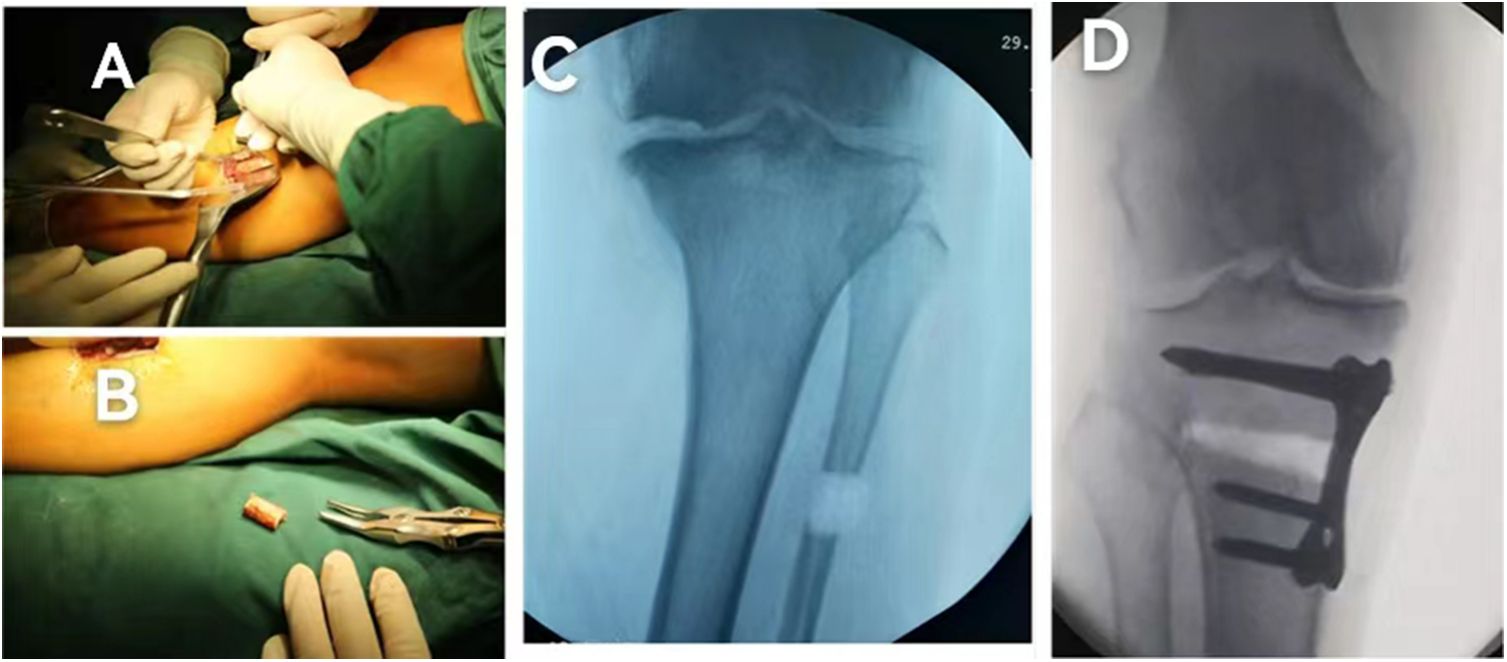
Open-wedge high tibial osteotomy and proximal fibular osteotomy. (**A**) Exposure of the fibula, (**B**) the 1.5-cm fibula is cut off with a pendulum saw, (**C**) the intraoperative radiograph of proximal fibular osteotomy, and (**D**) the osteotomy is fixed with a four-point support plate.

First, a proximal fibula osteotomy was conducted. A fibula posterolateral approach was adopted between the posterior and lateral muscles of the lower leg. A 3-cm longitudinal incision was made at the posterolateral side of the leg, 6–8 cm below the fibulae capitulum. The fibula was exposed through the gap between the peroneus longus and soleus muscles. The perifibular soft tissue was separated by a periosteal detacher. Under the protection of two bread boards, the 1.5 cm section of the fibula was cut off with a pendulum saw, and wrapped in a wet gauze (Figures 1A–C). After the OW-HTO was performed, the osteotomy was fixed with a four-point support plate (Figure 1D). The plate is from Shandong Weigao Orthopedic Material Co., Ltd. Its quality management system complies with GB/T 19001-2016 idt ISO 9001:2015. And the target postoperative mechanical axis was set as passing through 62.5% from the medial side of the tibial condyle (23).

**Figure 2:**
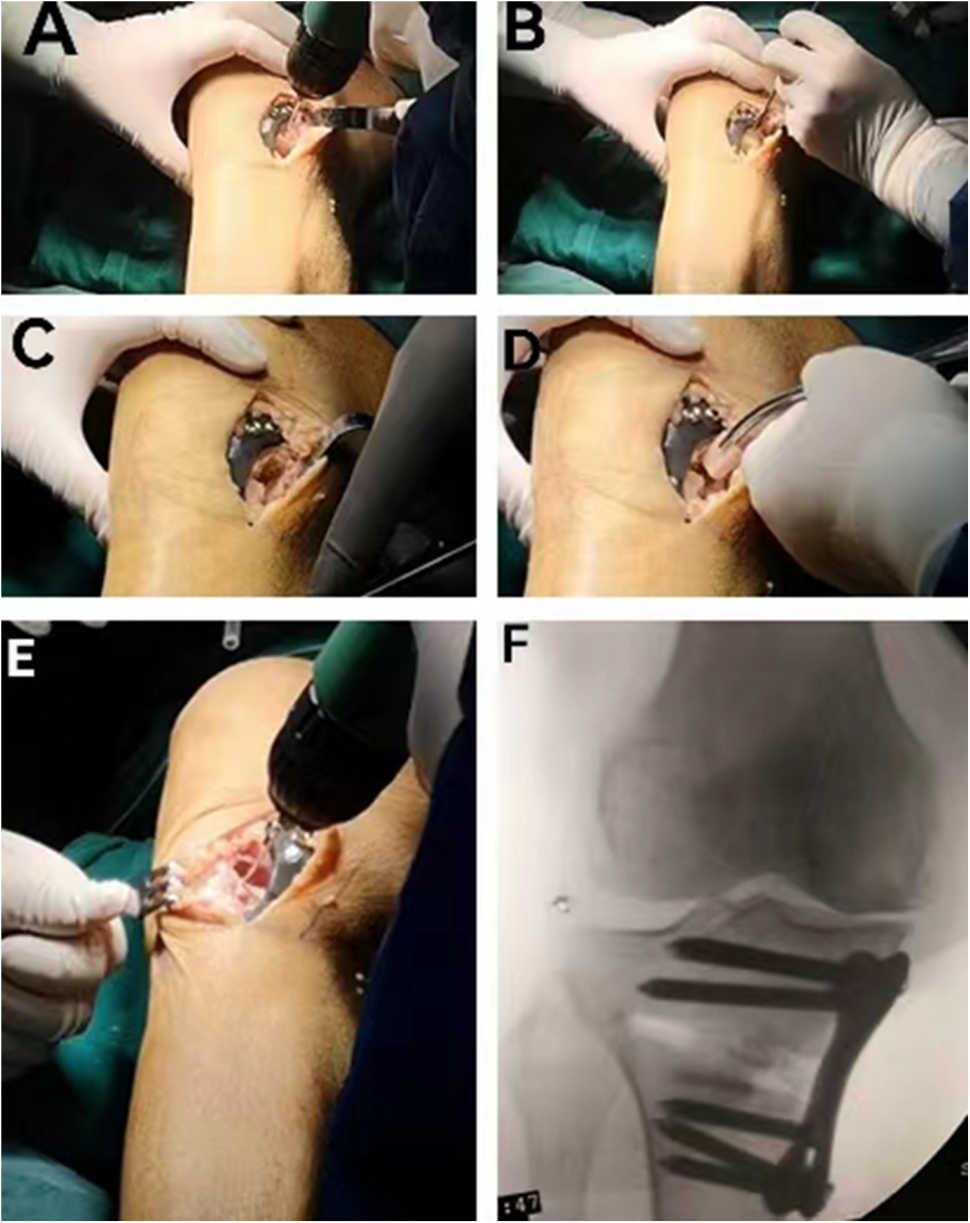
The procedure of the OBFT-FT. (**A**) A Kirschner-wire 1.0 mm in diameter was used to conduct the U-shaped osteotomy, (**B**) the remaining bone tissue was chiseled out with an osteotome for about 6 mm, (**C**) the orthotopic bone flap was performed, (**D**) half of the fibula was implanted into the gap, (**E**) contralateral U-shaped osteotomy, and (**F**) the intraoperative radiograph after OBFT-FT.

Lastly, the orthotopic bone flap transplantation and fibula transplantation were performed on both sides of the osteotomy gap. A Kirschner-wire 1.0 mm in diameter was used for a U-shaped osteotomy of the tibia at the upper edge of the osteotomy gap below the plate, with an area of 0.5 cm × 1.0 cm and a height 1.5 cm (Figure 2A). The remaining bone tissue was chiseled out with an osteotome for about 6 mm and the orthotopic bone flap was pushed into the osteotomy gap (Figures 2B,C), then half of the fibula was implanted into the gap left by the orthotopic bone flap (Figure 2D). As mentioned previously, the U-shaped osteotomy was performed on the tibia at the lower edge of the osteotomy space above the plate, and the remaining fibula was implanted (Figures 2E,F).

### Postoperative rehabilitation

All patients were encouraged to begin tolerable range of activities and passive motion and muscle strengthening exercises on the day after operation. Partial weight-bearing with the help of a walker was permitted for 1 week post-operatively. The time to full weight-bearing was dependent upon the patient's tolerance for weight-bearing activities after the sixth week.

### Data collection

**Figure 3:**
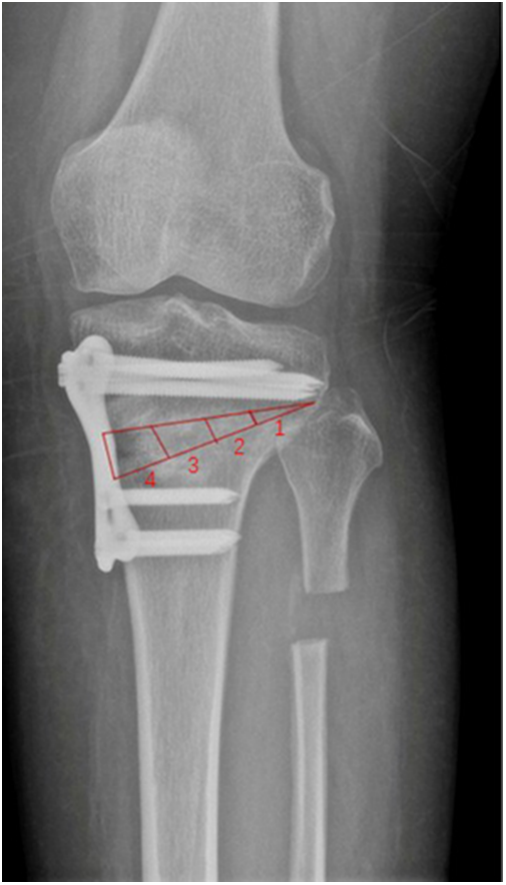
The osteotomy length was divided into four zones on the anteroposterior radiograph. When the bridging callus was more than half of the osteotomy gap (zone 2), it was called radiological bone union.

Follow-up visits at the outpatient department were scheduled at the following intervals: sixth week, third month, and sixth month post-operatively. Radiographic and functional outcomes were evaluated. Radiographic outcomes were determined using anteroposterior and lateral radiograph of the knee. Osteotomy filling index (OFI) was used to evaluate gap healing required according to Brosset et al. (10). OFI was assessed by dividing the osteotomy length into four zones (Figure 3). When the bridging callus was more than half of the osteotomy gap (zone 2), it was called radiological bone union. Clinical evaluation was performed using International Knee Documentation Committee (IKDC), Lysholm score, and visual analog scale (VAS) preoperatively and at the sixth-month follow-up.

### Statistical analysis

The data of clinical outcomes were analyzed by SPSS 23.0 software. Continuous variables were shown as the means ± SD. Student's *t* test or Mann–Whitney *U* test was employed to compare the difference between preoperative and postoperative data. And, statistical significance was defined as *p* < 0.05.

## Results

The ratios of radiological bone union during follow-up are provided in Table 2. Radiological gap healing was observed in six patients (42.9%) at 6 weeks after surgery, and that ratio increased to 85.7% (12 cases) at 3 months. All patients presented with radiological bone union at the 6-month follow-up. The functional outcomes at preoperative and at the sixth-month follow-up are provided in Table 3. The mean Lysholm Score, IKDC Score, and VAS scores at sixth-month follow-up were significantly better than those taken preoperatively (*p* < 0.001, *p* < 0.001, *p* = 0.001, respectively). And no delayed union or non-union, collapse, loss of correction, and surgical site infection were found.

**Table 2:** Ratio of radiological gap healing during follow-up.

Follow-up visit	Radiological gap healing, *n* (%)
6 weeks	6 (42.9)
3 months	12 (85.7)
6 months	14 (100)

**Table 3:** Functional outcomes.

Variables	Preoperative	6 months	*P*-value
Lysholm score, mean ± SD	59.2 ± 8.3	82.4 ± 6.3	<0.001
IKDC score	44.9 ± 9.6	69.2 ± 8.2	<0.001
VAS scores, mean ± SD	4.9 ± 1.7	2.9 ± 1.5	0.001

## Discussion

Osteotomy gap is a major disadvantage of OW-HTO as it causes different complications. Some surgeons prefer to keep it vacant, and the others tend to fill it with various materials. However, it has been reported that the excellent management of the osteotomy gap could facilitate the gap healing after OW-HTO to help recover initial mechanical stability and permit early weight-bearing, which are vital for the prevention of hardware failure, delayed or non-union, surgical incision infection, deep vein thrombosis, and loss of correction (7, 24–26). Therefore, various operative techniques and gap fillers have been adopted to accelerate gap healing after OW-HTO. In the present study, OBFT-FT as one new technique of autologous bone graft was used to promote the gap healing after HTO. Satisfactory results in all patients were observed in terms of both radiological outcomes and functional outcomes.

The process of osteotomy gap healing occurs from the lateral hinge to medial cortex of the tibia after OW-HTO. radiological bone union is assessed by the visible callus achieving 50%–75% (zone 3) of the osteotomy gap according to Brosset et al. (10) Although no consensus has been reported on the time of gap union in previous studies, most studies insisted that average healing time was >3 months (7, 9). Jung et al. reported that the mean time of radiological bone union in patients without gap fillers was 7.2 ± 3.2 months according to their studies (8). Staubli and Jacob reported that 6–18 months were required for the new bone filling in 75% of the gap (27). While Staubli and Jacob reported that more than 75% of the gap filling with the new bone occurred within 6 months in their study. In addition, the healing of the osteotomy gap is affected by the size of the gap, and larger correction angles need more time to heal. Fucentese et al. observed that patients with smaller gap sizes demonstrated a higher degree of bone union, which improved over time (31). Furthermore, the study of Goshima et al. has shown that the large osteotomy gap was an independent risk factor for the delayed bone healing after OW-HTO (28). Delayed union and non-union are disastrous complications after OW-HTO, which could be seen in 6.6%–15% and 1.6%–7.0% of cases, respectively (10, 28–30). Fucentese et al. reported that gap filler was a significant accelerating factor for bone union in their study (31). Meanwhile, bone grafting has been actively recommended in OW-HTO for preventing delayed union and non-union, especially for patients with correction angles >10° (9, 32).

Autograft is identified as the gold standard for the filling of osteotomy gaps (12). The most common material of autologous bone graft is the iliac crest graft, the other materials include distal femoral cancellous bone graft and autologous osteophyte graft (31, 33, 34). Autologous iliac crest graft can provide osteogenesis properties and structural support, the potential negative comorbidities and additional intraoperative operations make it controversial. Moyad and Minas et al. reported the technique of distal femoral cancellous bone graft, in which a cylindrical autologous bone graft was extracted from medial femoral condyle at the level of adductor tubercle (35). Jung et al. combined the distal femoral cancellous bone graft and β-tricalcium phosphate to promote the healing of osteotomy gap after OW-HTO, and satisfactory radiological and clinical outcomes were observed (8). In view of the limited studies on the application of distal femoral cancellous bone graft in OW-HTO, the safety, efficiency, and mid- or long-term effects on the femoral condyle should be further observed. Akiyama et al. proposed the technique of autologous osteophyte graft, which was only applied in 11 patients with OW-HTO (34). The autologous osteophyte was harvested from the knee by arthroscopy. The potential risks of damaging the cruciate ligament, meniscus, and cartilage are its major disadvantages.

Compared with the above autologous bone techniques, OBFT-FT has the following advantages: (a) the operation of OBFT-FT is simple, and it has a short learning curve. Both orthotopic bone flap technique and proximal fibular osteotomy are easy to operate. Proximal fibular osteotomy is accompanied with fewer complications and is easily executed. The operative approach passing through the gap between the peroneus longus and soleus muscles is intuitive, and there are no important nerves, blood vessels around. (b) It provides excellent osteogenic properties and stable structural support. The orthotopic bone flap can exist as a framework for bony ingrowth. And the abundant cancellous bone provides osteoprogenitor cells, bone morphogenetic proteins, and growth factors, which can promote the formation of new bone. The removed fibula belongs to the cortical bone, the transplantation of which is conducive to keep the mechanical stability. (c) It can avoid most of the potential donor site morbidities in autograft. The major disadvantages of autologous iliac crest graft are donor site morbidities, including hematoma, seroma, postoperative pain, dysesthesia, surgical incision infection, vascular injury, and iliac crest fracture (36). However, the additional donor site is not required in OBFT-FT. With a simple operation and intuitive surgical approach, the complications of proximal fibular osteotomy are extremely rare. And no relative complication of the OBFT-FT was discovered in the present study. (d) It does not increase the economic burden of patients.

In this study, the mean gap size was 10.2 ± 2.7 mm, which was similar to the findings of Slevin et al. (7). They reviewed 1,421 patients with OW-HTO and found a mean gap size of 9.8 mm. And the present study showed an excellent radiological gap healing rate at the sixth-week and third-month follow-ups (42.9% and 85.7%, respectively). The process of gap healing in our study is faster than in previous studies. Park et al. reported that the rates of radiological gap healing without gap fillers at 6 weeks and 3 months were 22.7% and 74.6% in their study (9). Siboni et al. reported 87.8% of cases showed bone union at a mean of 5 ± 3 months (37). Fucentese et al. reported that the rate of osseous gap healing was 40.1% at the third month (31). In addition, all of the patients who were observed showed good clinical outcomes during the sixth-month follow-up period, which was consistent with previous studies (31, 8). In the present study, the Lysholm score, IKDC score, and VAS scores improved significantly (*p* < 0.001, *p* < 0.001, *p* = 0.001, respectively) at the sixth-month follow-up when compared with those prior to the operation.

### Limitation

There were several limitations in this study. First, it was a retrospective, non-randomized control trial, and non-comparative study. But we proposed a new autograft technique for OW-HTO, and we observed its efficiency by radiological and clinical evaluation, which could help us master the short-term outcomes of this technique. Second, the sample size was limited. Third, the degree of gap healing was evaluated by anteroposterior radiographs. The two- dimensional radiographs could not accurately reflect the gap healing in three dimensions. Computed tomography is an ideal method to evaluate the degree of gap healing without increasing the patient's radiation. In the future, the studies on OW-HTO with or without OBFT-FT or autogenous iliac graft will be conducted.

## Conclusion

As a new technique for autologous bone graft, the OBFT-FT could be successfully applied in the treatment of gap healing after OW-HTO, and excellent radiological and clinical outcomes could be seen in short-term follow-up.

## Data Availability

The raw data supporting the conclusions of this article will be made available by the authors, without undue reservation.
