# Supplementary material for: A new technique of autologous bone grafting for open-wedge high tibial osteotomy
Source: Front Surg. 2024 Mar 5;11:1337668. doi: 10.3389/fsurg.2024.1337668 (PMC10948400; doi:10.3389/fsurg.2024.1337668)
Supplement: Supplementary file 1 [file Table1.docx]

**Table 1**

Patient demographic data and radiographic data.

| variables | Number |
| --- | --- |
| Age(years), mean ± SD | 59.6 ± 9.2 |
| Gender (male), no. (%) | 3(21.4) |
| Bmi, mean ± SD | 28.1 ± 4.5 |
| Correction angle(deg), mean ± SD | 9.5 ± 1.8 |
| Gap width(mm), mean ± SD | 10.2 ± 2.7 |
| Lateral hinge fracture, n (%) | 1(7.1) |

abbreviation: SD, standard deviation; Body Mass Index, Bmi.
