# Supplementary material for: A new technique of autologous bone grafting for open-wedge high tibial osteotomy
Source: Front Surg. 2024 Mar 5;11:1337668. doi: 10.3389/fsurg.2024.1337668 (PMC10948400; doi:10.3389/fsurg.2024.1337668)
Supplement: Supplementary file 2 [file Table2.docx]

**Table 2**

Ratio of radiological gap healing during follow-up.

| follow-up visit | radiological gap healing, n (%) |
| --- | --- |
| 6 weeks, | 6(42.9) |
| 3 months | 12(85.7) |
| 6 months | 14(100) |

abbreviation: SD, standard deviation.
