# Supplementary material for: A new technique of autologous bone grafting for open-wedge high tibial osteotomy
Source: Front Surg. 2024 Mar 5;11:1337668. doi: 10.3389/fsurg.2024.1337668 (PMC10948400; doi:10.3389/fsurg.2024.1337668)
Supplement: Supplementary file 3 [file Table3.docx]

**Table 3**

Functional outcomes.

| Variables | Preoperative | 6 months | P value |
| --- | --- | --- | --- |
| Lysholm Score, mean ± SD | 59.2 ± 8.3 | 82.4 ± 6.3 | <0.001 |
| IKDC Score | 44.9 ± 9.6 | 69.2 ± 8.2 | <0.001 |
| VAS scores, mean ± SD | 4.9 ± 1.7 | 2.9 ± 1.5 | 0.001 |

abbreviation: SD, standard deviation; IKDC, International Knee Documentation Committee; VAS, Visual Analogue Score.
